# A Substrate-Fusion Protein Is Trapped inside the Type III Secretion System Channel in *Shigella flexneri*

**DOI:** 10.1371/journal.ppat.1003881

**Published:** 2014-01-16

**Authors:** Kim Dohlich, Anna Brotcke Zumsteg, Christian Goosmann, Michael Kolbe

**Affiliations:** 1 Structural Systems Biology Group, Max-Planck-Institute for Infection Biology, Berlin, Germany; 2 Department of Cellular Microbiology, Max-Planck-Institute for Infection Biology, Berlin, Germany; 3 Microscopy Core Facility, Max-Planck-Institute for Infection Biology, Berlin, Germany; Stanford University School of Medicine, United States of America

## Abstract

The Type III Secretion System (T3SS) is a macromolecular complex used by Gram-negative bacteria to secrete effector proteins from the cytoplasm across the bacterial envelope in a single step. For many pathogens, the T3SS is an essential virulence factor that enables the bacteria to interact with and manipulate their respective host. A characteristic structural feature of the T3SS is the needle complex (NC). The NC resembles a syringe with a basal body spanning both bacterial membranes and a long needle-like structure that protrudes from the bacterium. Based on the paradigm of a syringe-like mechanism, it is generally assumed that effectors and translocators are unfolded and secreted from the bacterial cytoplasm through the basal body and needle channel. Despite extensive research on T3SS, this hypothesis lacks experimental evidence and the mechanism of secretion is not fully understood. In order to elucidate details of the T3SS secretion mechanism, we generated fusion proteins consisting of a T3SS substrate and a bulky protein containing a knotted motif. Because the knot cannot be unfolded, these fusions are accepted as T3SS substrates but remain inside the NC channel and obstruct the T3SS. To our knowledge, this is the first time substrate fusions have been visualized together with isolated NCs and we demonstrate that substrate proteins are secreted directly through the channel with their N-terminus first. The channel physically encloses the fusion protein and shields it from a protease and chemical modifications. Our results corroborate an elementary understanding of how the T3SS works and provide a powerful tool for *in situ*-structural investigations in the future. This approach might also be applicable to other protein secretion systems that require unfolding of their substrates prior to secretion.

## Author Summary

Type III Secretion Systems (T3SS) secrete bacterial effector proteins from the cytoplasm across the cell wall, but mechanistic details of this process remain mostly elusive. We locked the T3SS of *Shigella flexneri* in an actively secreting state by expression of substrate fusions that consist of a functional translocator and a stably-folded knotted protein. Although recognized as T3SS substrates, the fusions are not released from secreting *Shigella* and impede transport of other effector proteins by obstructing the T3SS channel. We localized the fusion at isolated channels and observed that the translocator is secreted with the N-terminus first. We further demonstrate that the channel physically encloses the partially transported substrate. Our analysis elucidates important steps of the T3SS mechanism. Furthermore, we developed fusion proteins useful for advanced structural investigations of one of the most complex bacterial virulence devices known and our approach may help to also understand other protein transport mechanisms.

## Introduction

T3SS are found in numerous Gram-negative bacteria and share strong homologies among different invasive pathogens. Using the T3SS, bacteria are able to secrete effector proteins that translocate into the host-cell where they target metabolic or signal transduction pathways for example [1] [2]. The T3SS is a key instrument in interactions between bacteria and eukaryotes, as it is used also by symbiotic bacteria in plants [3] [4].

One pathogen depending on T3SS-mediated virulence is *Shigella flexneri*, a human pathogen of the intestine that depends on effector protein delivery to establish an infection. In *S. flexneri* serovar 5a M90T, the T3SS is encoded on a 210 kb-extrachromosomal plasmid [5], where genes encoding the NC are clustered in distinct operons. About 25 genes that lie in the membrane-expression of invasion plasmid antigen (*mxi*)-locus and surface presentation of antigen (*spa*)-locus constitute the NC. Together with the invasion plasmid antigen (*ipa*)-operon, this region is referred to as the entry region which is necessary and sufficient for invasion of host cells [6] [7]. The translocators IpaB and IpaD regulate secretion by forming a complex at the tip of the needle [8] and deletion of either *ipaB* or *ipaD* causes hypersecretion of effectors [9].

As for other bacterial T3SS [10] [11], the NC from *S. flexneri* shows striking structural similarity to a syringe with a basal body and a needle-like hollow tube that can be isolated from the bacterial envelope [12]. The basal body is made of stacked protein rings that are inserted into the inner and outer membrane. Together, these rings form a conduit which narrows into the needle that protrudes from the bacterium. The needle is made of many copies of one small subunit protein that assembles into a helical tube. Both the basal body and the needle form a continuous channel that ranges from the bacterial cytoplasm to the extracellular environment. The inner diameter of the needle channel was estimated to be 2–3 nm [12] [13], and recent structural analysis defined a 2.5 nm channel in *Salmonella enterica* serovar Typhimurium SPI-1 with a conserved architecture in *S. flexneri* [14] [15].

Type III secretion mechanism has been studied using fusions of effectors and stably folded protein domains [16] [17] [18] [19] [20]. For example, fusion proteins consisting of *Yersinia enterocolitica* effectors fused to dihydrofolate reductase (DHFR) or ubiquitin either obstructed the T3SS [17] [18] [19] or were rejected [18]. If the fold of DHFR or ubiquitin was destabilized by mutations or by the action of a chaperone, fusions were readily secreted by the T3SS [17] [18]. This implies that fused effectors can be secreted by the T3SS if the substrate is unfolded prior to secretion. Taken together, whether or not a T3SS substrate is compliant for secretion through the NC channel seems to depend on its fold and structural stability. These data support the hypothesis that effector proteins need to be unfolded in order to be efficiently secreted through the needle channel.

While the model of secretion through the NC channel has been widely accepted, no experimental evidence for this model exists [21] [22]. Furthermore, the whole idea of the T3SS working as a microsyringe injecting effectors into the host cell has been questioned [22]. A substrate has neither been pictured in contact with the NC nor has an actively secreting NC been used for structural investigations. Therefore, our strategy was to trap a substrate inside the *S. flexneri* NC, using a fusion protein which cannot be unfolded by the T3SS. We designed fusion proteins that consist of the translocator IpaB and the RNA 2′-O-ribose methyltransferase RrmA (PDB ID 1IPA). RrmA has a trefoil-knot in its C-terminal region [23] and we will refer to RrmA as “Knot”. Here, we present a direct visualization of the NC together with IpaB-Knot. We show that the NC channel physically encloses its substrate and experimentally confirm a substantial hypothesis of the T3SS secretion mechanism. To our knowledge, this is the first demonstration of a substrate being transported through the NC channel.

## Results

### IpaB-Knot is functional and folded

**Figure 1:**
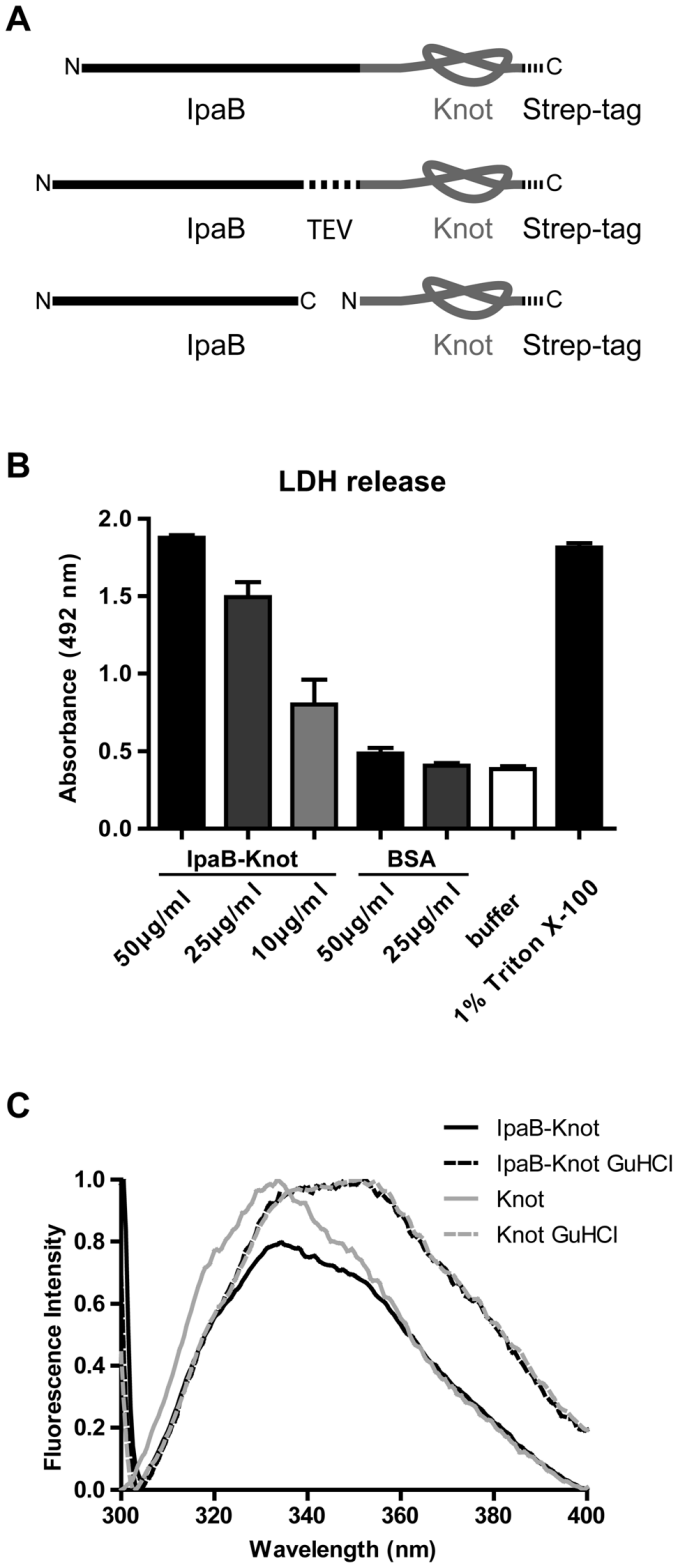
IpaB-Knot fusions and functional analysis. (*A*) Schematic of the fusion protein consisting of virulence factor IpaB and the knot-forming domain Knot with a C-terminal Strep-tag. Second construct with an embedded TEV-protease cleavage site inbetween IpaB and the Knot. Third construct of Knot separated from IpaB. (*B*) Murine bone marrow macrophages were treated with different concentrations of IpaB-Knot. LDH release was measured by absorption in a colorimetric assay as a marker for cellular disintegration. Error bars indicate SEM, n = 3 independent experiments. (*C*) Tryptophan fluorescence of IpaB-Knot compared to Knot under native (solid lines) and denaturing (dashed lines) conditions in the presence of 6 M Guanidine HCl. Fluorescence intensity was normalized to 1.

IpaB is a multifunctional protein that induces pyroptosis in macrophages by lysosomal leakage and activation of Caspase-1 [24] [25]. We constructed translational fusions consisting of IpaB followed by the Knot (IpaB-Knot) with either a leucine-glutamine linker, a TEV protease site between both proteins or the Knot expressed without IpaB-fusion (Fig. 1*A*). To investigate whether IpaB was still functional when fused to the Knot, induction of pyroptosis by purified IpaB-Knot was quantified in a cytotoxicity assay. Induction of pyroptosis was measured by LDH released from murine bone marrow-derived macrophages (mBMM) that were treated with increasing concentrations of IpaB-Knot (Fig. 1*B*). The fusion protein used in this assay was free of contaminants and wildtype IpaB (e.g. resulting from fusion protein instability) which could give false positive results (Fig. S1). We observed a similar dose-dependent release of LDH with IpaB-Knot as has been demonstrated for IpaB alone [25]. 50 µg/ml of recombinant fusion protein caused LDH release as efficiently as 1% detergent (Triton X-100). These results demonstrate that IpaB is still functional when fused to the Knot.

We also addressed the folding status of the Knot-domain in IpaB-Knot by measuring the change in tryptophan fluorescence upon unfolding (Fig. 1*C*). IpaB contains a single tryptophan whereas the Knot domain has four tryptophans, three of which are part of the trefoil-knot in the C-terminal region. This indicates that the fluorescence emission spectrum mainly represents the folding status of the Knot. We compared spectra of purified IpaB-Knot and the Knot alone, either in buffer or in the presence of the chaotropic salt Guanidine HCl (GuHCl). The native Knot protein has a fluorescence emission peak at 335 nm which represents tryptophans in the folded protein (Fig. 1*C*, solid grey line). The fluorescence spectrum of IpaB-Knot is similar to the native Knot spectrum with an emission peak at 335 nm (Fig. 1*C*, solid black line), suggesting that the Knot is folded when fused to IpaB. Protein unfolding in the presence of 6 M GuHCl leads to a red shift of the emission peak from 335 nm to 355 nm which indicates a change of the molecular environment of the respective tryptophans in both IpaB-Knot and Knot alone. This change in fluorescence is caused by structural changes of the protein and demonstrates that the Knot domain was folded in both cases and is sensitive to unfolding by GuHCl.

The analysis of folded parts of the Knot domain in IpaB-Knot was in line with these findings. Protein fragments were obtained by treatment with the non-specific protease Proteinase K. Protein fragments protected from cleavage by their native fold were separated by 2D-gel electrophoresis and further analyzed by mass spectrometry (MS) according to the procedure described by Jungblut *et al.* [26] [27]. We detected polypeptides that cover almost the entire sequence of the Knot domain and the complete sequence of the trefoil-knot motif. Subfragments were confirmed by MS/MS analysis (Fig. S2). Only the C-terminal helix and N-terminal parts of the Knot domain, but not the trefoil-knot, were cleaved, suggesting that this motif is folded and consequently inaccessible to Proteinase K. Together with our findings from fluorescence analysis, these results confirm a tightly folded core domain covering the trefoil knot-motif in IpaB-Knot which is inaccessible to Proteinase K and sensitive to unfolding by the chaotropic agent GuHCl.

### IpaB-Knot attenuates invasion of M90T

IpaB is essential during *S. flexneri* invasion of epithelial cells [7]. Since IpaB-Knot is functional, we analyzed whether bacteria with a genomic *ipaBknot* fusion allele were still invasive compared to the wildtype. The fusion *ipaBknot* was introduced by insertion of the *knot*-gene (together with a 3′ *strep*-tag) downstream of the *ipaB*-open reading frame in *S. flexneri* M90T. Hence, *ipaBknot* was expressed under the native promoter of the *ipgCipaBCDA*-operon (M90T::*ipaBknot*).

**Figure 2:**
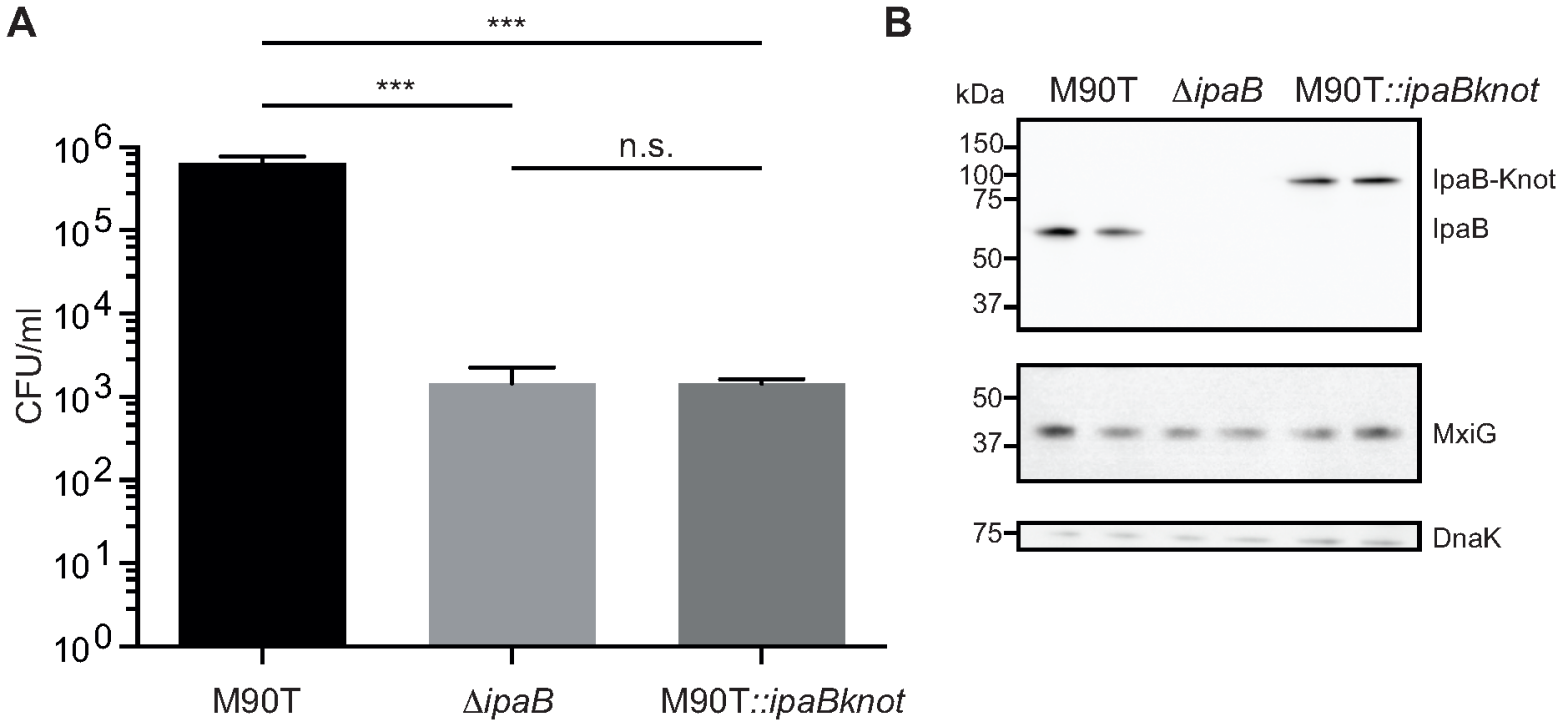
Invasion is attenuated in M90T carrying the fusion allele. (*A*) Invasion assay with wildtype *S. flexneri* M90T, the non-invasive Δ*ipaB* and M90T with the fusion allele(M90T::*ipaBknot*, quantified by colony forming-units (CFU) per ml of culture. Error bars indicate standard deviation, performed as duplicates (bacterial clones), n = 3 technical replicates, analysis by multiple t-tests. (*B*) Western blot analysis of IpaB (anti-IpaB mouse monoclonal), MxiG as a marker for NCs (anti-MxiG mouse monoclonal), DnaK as a loading control (anti-DnaK mouse monoclonal).

M90T::*ipaBknot* was compared to wildtype M90T and the invasion-deficient 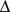 *ipaB* strain. Invasiveness was quantified in a gentamicin protection assay [28] where bacteria are allowed to replicate inside host cells after invasion. Clearly, M90T::*ipaBknot* is attenuated in invasion and is not significantly different from the negative control 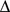 *ipaB* (Fig. 2*A*). This attenuation does not result from altered expression of IpaB-Knot compared to IpaB in M90T (Fig. 2*B*) or a deficit in expressing structural proteins of the NC. Levels of IpaB are equal in all strains tested as were levels of MxiG which constitutes the inner membrane ring, an integral part of the needle complex [12]. As we have already demonstrated that IpaB is functional in IpaB-Knot, the non-invasive phenotype might result from effects on the T3SS pathway.

### Substrate-Knot fusions impede type III secretion

**Figure 3:**
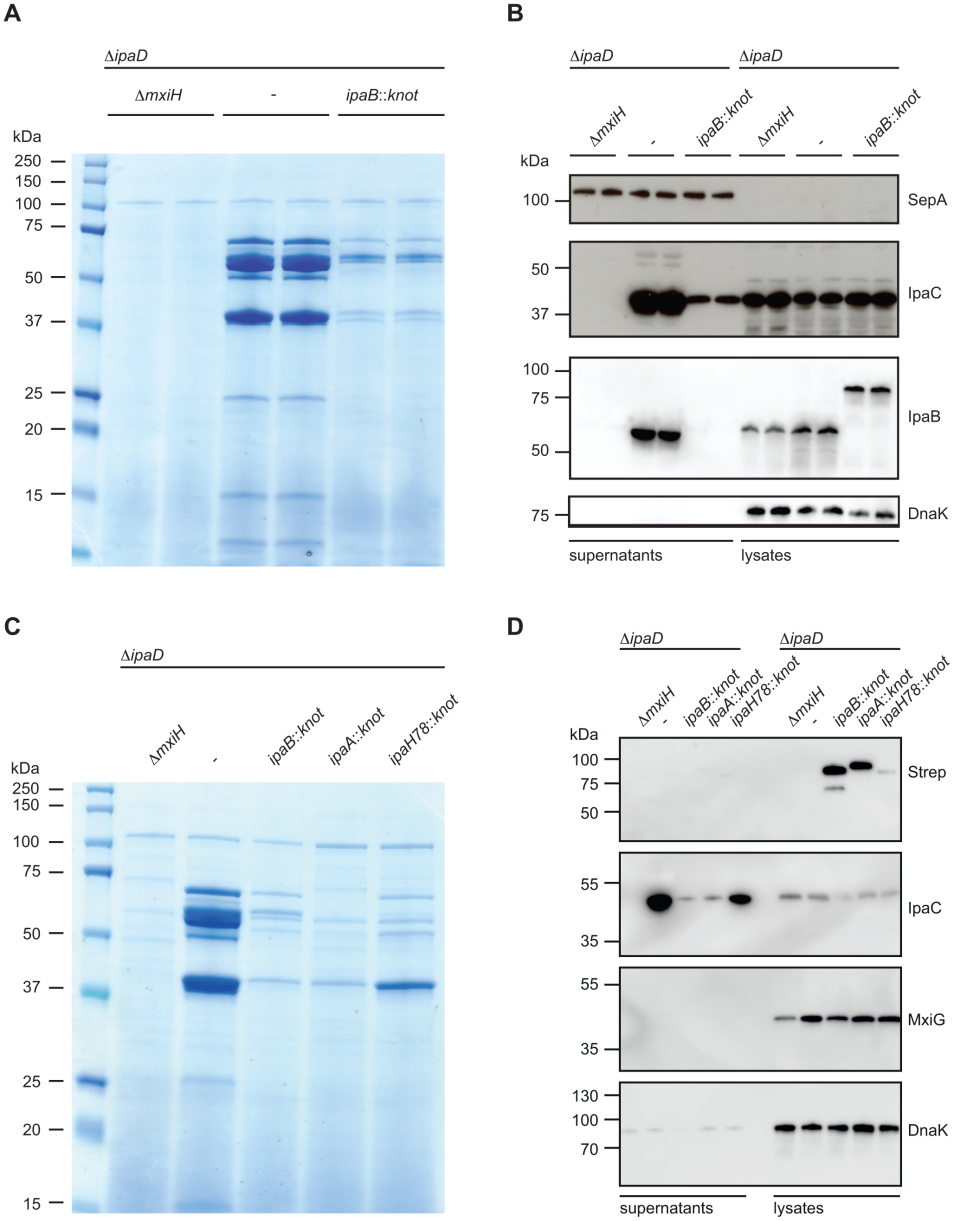
Substrate-Knot effects on secretion in Δ*ipaD*. (*A*) SDS PAGE/Coomassie staining of precipitated supernatants from overnight-grown TSB cultures. Samples were analyzed in duplicate and normalized to OD 2/ml. (*B*) Western Blot analysis of SepA (T3SS-independent protein), IpaC, IpaB and DnaK (intracellular chaperone/lysis control) in supernatants (left) and whole-cell lysates (right). (*C*) SDS PAGE/Coomassie staining of precipitated culture supernatants from controls, ipaB- and effector-fusion strains. Samples normalized to OD 2/ml. (*D*) Western Blot analysis from samples from (*C*) detecting Strep-tag (fusion protein expression marker), IpaC, and DnaK (intracellular chaperone/lysis control) in supernatants (left) and whole-cell lysates (right).

In order to elucidate effects on secretion, we inserted the functional fusion encoding *ipaBknot* in the virulence plasmid of an *ipaD*-deficient strain (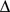 *ipaD*::*ipaBknot*). The 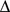 *ipaD* strain secretes IpaB as well as other effectors without the need for T3SS induction [9]. Therefore, we compared protein secretion in 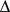 *ipaD*, 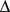 *ipaD*::*ipaBknot*, and a secretion-deficient negative control (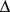 *mxiH* 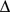 *ipaD*). Secreted proteins were analyzed by Coomassie stain of SDS PAGE (Fig. 3*A*) and Western blot (Fig. 3*B*). Supernatants from the 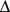 *ipaD* strain show a pattern of various proteins secreted. In contrast, secretion of proteins in the 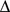 *ipaD*::*ipaBknot* strain was dramatically reduced. Attenuation of secretion was limited to T3SS-dependent proteins, since secretion of SepA, a substrate to a type V secretion system [29], was not influenced by IpaB-Knot (Fig. 3*A*, 110 kDa and 3*B*, upper panel).

Western blot analysis supported our findings of limited secretion in the 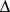 *ipaD*::*ipaBknot* strain (Fig. 3*B*). We probed for the translocator IpaC in supernatants and bacterial lysates from 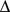 *ipaD* and 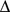 *ipaD*::*ipaBknot*. Reduction of secreted IpaC in supernatants was not due to a diminished synthesis, as intracellular levels of the protein were equal in all three strains. Importantly, IpaB was secreted by the 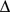 *ipaD* mutant whereas IpaB-Knot was only detected in bacterial lysates of 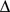 *ipaD*::*ipaBknot*, indicating its cytoplasmic localization (Fig. 3*B*). We conclude that fusing the knot to IpaB prevents secretion of IpaB itself and impedes secretion of other T3SS effectors.

We wondered whether additional effector-knot fusions could block the T3SS, or if the phenotype we observed was limited to IpaB-Knot. Therefore, we fused the knot to the early secreted effector IpaA and the late secreted effector IpaH7.8 [30]. As seen with IpaB-Knot, IpaA-Knot and IpaH7.8-Knot fusions dramatically reduced secretion of effectors (Fig. 3*C*. We further delineated secretion by Western blot analysis and found that IpaA-Knot and IpaH7.8-Knot remained cytosolic while blocking secretion of other effector proteins (Fig. 3D). The overall reduction in secretion was not due to a lack of T3SS needle complexes as the expression of T3SS structural components, indicated by MxiG, was not altered by expression of substrate-knot fusions. Interestingly, secretion of IpaC was attenuated in Δ*ipaD*::*ipaBknot* and 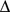 *ipaD*::*ipaAknot* strains but not in the late effector-fusion allele 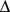 *ipaD*::*ipaH7.8knot*. As secretion of early effectors is a prerequisite for transcription of late effectors, this likely reflects the hierarchy of secretion that is characteristic of *Shigella*. Taken together these results suggest that effector-Knot fusions can be used as a general tool to obstruct T3SS.

As a control, the Knot alone was expressed by insertion of the *knot*-encoding gene at the same locus without fusion of the *ipaB*-gene (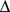 *ipaD*::*knot*, Fig. 1*A*). *knot* was inserted with a stop-codon for *ipaB* together with the *ipaC* ribosomal binding site mediating translation. In these experiments, secretion of T3SS effectors from 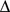 *ipaD*::*knot* were the same as for 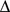 *ipaD* (Fig. S3 *A*). We found that expression of the *knot* alone did not affect the T3SS pathway, including effector synthesis, nor did it exert toxicity, as IpaB and MxiG were equal to 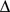 *ipaD* (Fig. S3 *B*). In summary, only the fusion of IpaB to the Knot reduced effector secretion by the T3SS in the otherwise hypersecreting 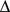 *ipaD* strain.

### IpaB-Knot co-purifies with isolated T3SS needle complexes

**Figure 4:**
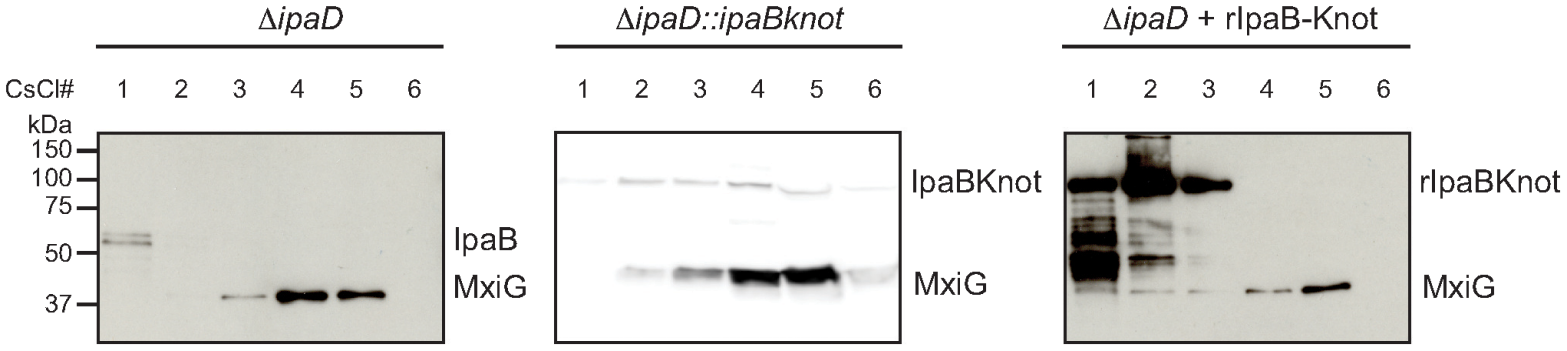
Co-localization of IpaB, IpaB-Knot and isolated NC. CsCl fractionation of NC from 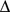 *ipaD* (left panel) and 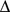 *ipaD*::*ipaBknot* (middle panel) or recombinant IpaB-Knot (rIpaB-Knot) (right panel). Samples were analyzed by Western blot with IpaB and MxiG antibodies.

Next, we studied the interaction of IpaB-Knot and NCs from 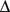 *ipaD*::*ipaBknot* by cesium chloride density fractionation. NCs were isolated from the bacterial membrane and separated from soluble proteins by centrifugation as it migrates to high-density fractions. Isolated NCs were detected with an anti-MxiG antibody. In line with the hypothesis of IpaB being unable to bind to the NC in the 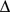 *ipaD* strain [8], residual IpaB was separated from the NCs. IpaB remained in the low-density fraction whereas NCs migrated to high-density fractions (Fig. 4, left panel, 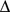 *ipaD*). In contrast, IpaB-Knot migrated with isolated NCs from 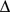 *ipaD*::*ipaBknot* to high-density fractions, which indicates an interaction with the NC (Fig. 4, middle panel, 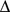 *ipaD*::*ipaBknot*). As a control, IpaB-Knot from recombinant expression (rIpaB-Knot) was added to purified NCs from 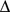 *ipaD* cells (Fig. 4, right panel, 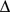 *ipaD*+rIpaBKnot). Purified IpaB-Knot remained in low-density fractions and did not migrate with NCs to high-density fractions. A co-migration was only observed for endogenous IpaB-Knot and not purified IpaB-Knot that was added to the NCs. Therefore, interaction of IpaB-Knot and the NCs from 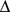 *ipaD*::*ipaBknot* most likely results from its attempted secretion and subsequent obstruction of the T3SS by IpaB-Knot.

### IpaB-Knot and the needle complex co-localize

**Figure 5:**
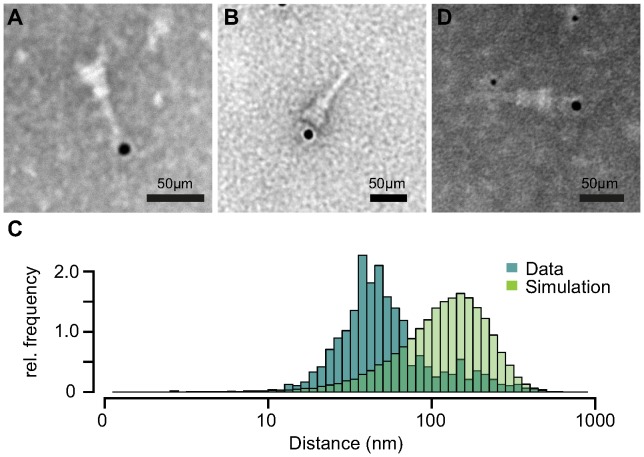
Immuno-electron microscopy of NCs and IpaB-Knot. Micrographs of NCs labeled with anti-IpaB antibody and gold-conjugated (12 nm) secondary antibody with IpaB localized at the NC tip (*A*). Strep-tag labeling with anti-Strep-antibody at the NC base (*B*). (*C*) Nearest-neighbor analysis of gold particles and isolated NCs. Relative counts of distances from 50 images are plotted (dark-green), together with random distributions of NCs and gold (light green) for each image. (*D*) Double-labeling with anti-IpaB antibody and gold-conjugated (6 nm) anti-human secondary antibody and anti-Strep antibody with 12 nm gold-conjugated anti-mouse secondary antibody. IpaB epitope localizes at the tip, Strep epitope at the basal side of the NC.

Based on our previous results, we tested if IpaB-Knot is arrested inside the NC channel as the Knot of the fusion remains folded and is impassable for the T3SS. Therefore, we analyzed the presence of IpaB-Knot with isolated NC channels after density fractionation using immuno-electron microscopy (iEM). Fractions that contained both IpaB-Knot and MxiG (Fig. 3, middle panel, fractions 4 and 5) were analyzed with an anti-IpaB monoclonal (H16) and an anti-Strep tag monoclonal antibody. The anti-IpaB monoclonal antibody recognizes an N-terminal stretch between the residues 118 and 179 [31]. The IpaB domain of IpaB-Knot was detected either at the needle tip or at the base of isolated NCs (Fig. 5*A* and Figure S4), whereas the C-terminal Strep-tag was only detected at NC bases (Fig. 5*B*). IpaB was not detected in samples of isolated NCs from the 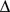 *ipaD* strain, which is consistent with our finding that IpaB does not co-purify in this background (Fig. 4, left panel). To assess whether the observed co-localization between IpaB-Knot and NC is specific, 50 random iEM images were quantified and coordinates for gold labels against IpaB and NC centers recorded. We found an average of 80 NC and 20 labels per image (Fig. S5). In order to obtain a random distribution of gold and NCs, respective coordinate X and Y values were randomized according to counts per image within the range of the image dimensions. Both counted or simulated coordinates were subjected to a nearest-neighbor analysis, quantifying distances between NC centers and the closest adjacent gold particles. The distribution of counted NC/gold distances peaks around 30–50 nm. In comparison, distances obtained from the random distribution peaked around 100 nm–120 nm.

We also labeled NCs with anti-IpaB and anti-Strep tag antibodies simultaneously. The anti-IpaB monoclonal antibody was expressed in a human cell line which resulted in a human Fc region. After labeling the NC with these primary antibodies, anti-human secondary antibody coupled to 6 nm gold and anti-mouse secondary antibody coupled to 12 nm gold were used. In a few cases, simultaneous labeling of both epitopes at the same NC was observed (Fig. 5*D*), with IpaB-labeling at the tip and Strep-labeling at the base. These results show co-localization of IpaB-Knot with the NC, resulting from a stable interaction between IpaB-Knot and NC.

### Protection of IpaB-Knot by the T3SS channel

We examined whether IpaB-Knot was inserted into the NC channel. According to the hypothesis that secretion of effectors and translocators occurs through the channel, part of IpaB-Knot should be enclosed and therefore be inaccessible to modifications.

**Figure 6:**
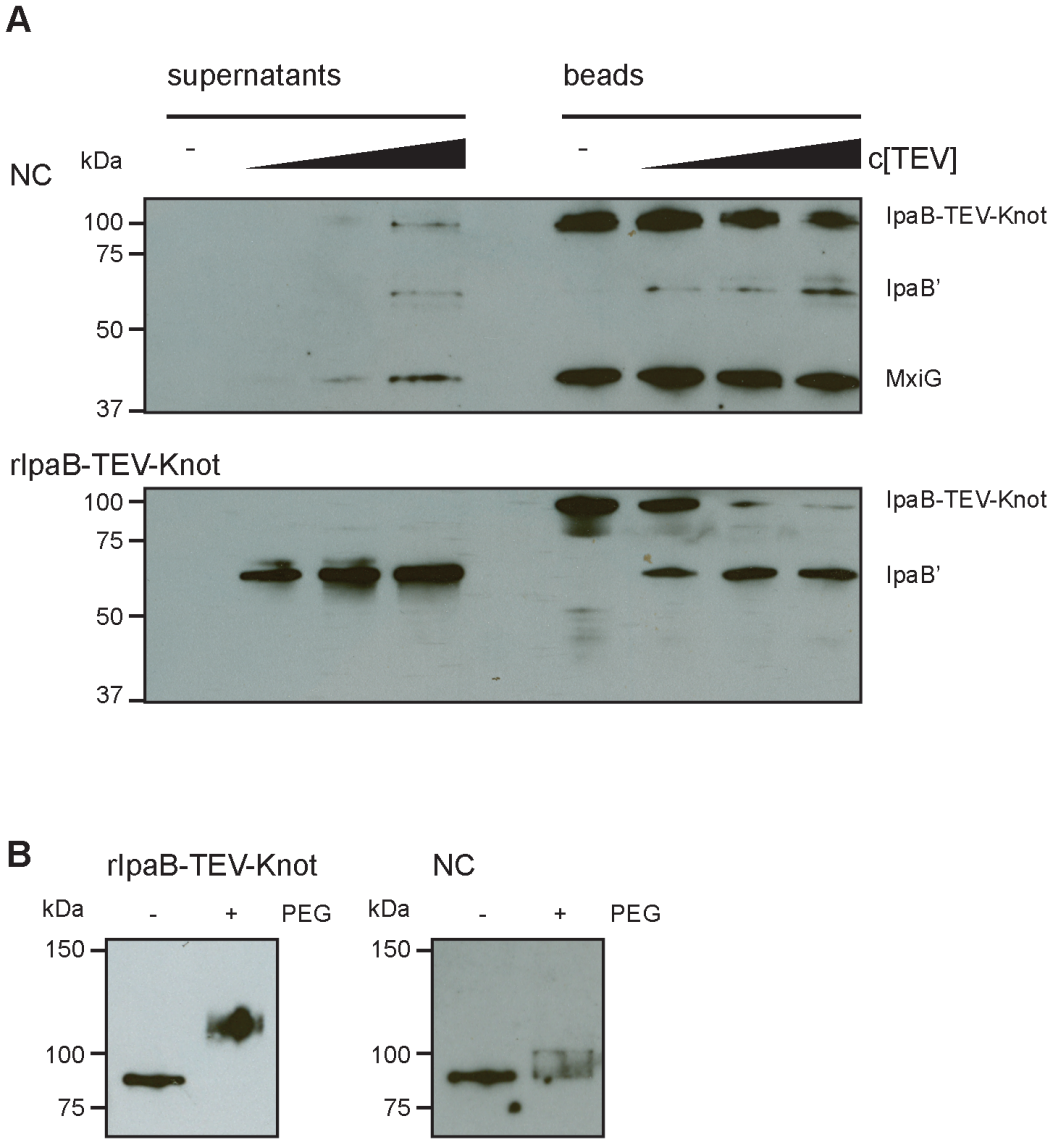
Protection and proteolysis of IpaB-TEV-Knot with isolated NCs and purified IpaB-TEV-Knot. (*A*) Sample supernatants and bead fractions from NC isolates from 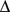 *ipaD*::*ipaBTEVknot* (upper panel) and purified IpaB-TEV-Knot (lower panel) were analyzed by Western blot with IpaB and MxiG antibodies after treatment with TEV protease. (*B*) Purified IpaB-TEV-Knot (left panel, rIpaB-TEV-Knot) or IpaB-TEV-Knot from NC isolates untreated (−) or treated with 0.5 mM MS(PEG)24 (+) analyzed by Western blot with Strep-tag antibody.

Our construct features a TEV protease-specific cleavage site between IpaB and the Knot domain together with a C-terminal Strep-tag (Fig. 1*A*). We would predict that, once IpaB-TEV-Knot is cleaved by TEV protease, IpaB' will be released from the beads into the sample supernatant. IpaB-TEV-Knot from 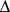 *ipaD*::*ipaBTEVknot* NC isolates and IpaB-TEV-Knot purified from *E. coli* BL21 were coupled to Strep-Tactin sepharose beads via the Strep-tag and subsequently treated with TEV protease. Both supernatants and bead fractions were analyzed by Western blot after protease treatment using IpaB and MxiG antibodies. IpaB-TEV-Knot co-purified with NCs showed almost no cleavage products (IpaB') after treatment with increasing concentrations of TEV protease (Fig. 6*A*, upper panel). The recombinant IpaB-TEV-Knot, however, was efficiently cleaved at low concentrations of TEV protease (Fig. 6*A*, lower panel). As IpaB-TEV-Knot decreased proportionally with increasing TEV protease concentrations, released IpaB' accumulated in supernatants and increased in direct correlation with the amount of protease. Some IpaB' attached to the beads after cleavage which could be due to a non-specific interaction between IpaB' and the beads. We conclude that IpaB-TEV-Knot co-purified with NC is largely protected from enzymatic cleavage by TEV protease. Cleavage of purified IpaB-TEV-Knot indicates an accessible TEV recognition site which was only observed for purified IpaB-TEV-Knot alone.

We also investigated whether IpaB-Knot can be modified by the addition of crosslinking PEG molecules (PEGylation). Each PEG adds 1.1 kDa to the proteins molecular weight and has an arm length of 8.8 nm and therefore cannot penetrate or diffuse into the NC channel for sterical reasons. PEGylation occurs via crosslinking of primary amines (lysine residues and N-terminus) to the NHS-group of PEG. IpaB-Knot has 58 PEGylation sites in total which are evenly distributed across the protein. Similar to the TEV protease assay, Strep-Tactin beads were saturated with either purified IpaB-Knot or isolated NCs containing IpaB-Knot and PEGylated. Samples were analyzed using a Strep-tag antibody since the tag has no PEGylation sites and the antibody epitope is not affected. We observed a size shift of PEGylated IpaB-Knot to about 110 kDa, which corresponds to PEGylation at 20–30 amines (Fig. 6*B*, left panel). On the other hand, PEGylation of IpaB-Knot isolated together with NCs occured to a lesser extent as the protein shifted not more than 10 kDa (Fig. 6*B*, right panel). We hypothesize that the difference is due to inaccessibility of amines resulting from the presence of the NC channel. Together with the results from the TEV protease-assay, we conclude that protection is conferred by the NC channel which surrounds IpaB-TEV-Knot and partially covers the protein.

## Discussion

In this study we demonstrate that T3SS substrate proteins travel through the NC channel during type III secretion. While largely assumed, our experiments provide convincing evidence for this model. Based on the strong conservation of the NC among different bacterial species [10] [11], we would predict that our findings are of general relevance for T3 secretion. We generated fusion proteins consisting of the translocator IpaB as a T3SS substrate and a protein with a trefoil-knot in its C-terminal region (Knot). We show that IpaB-Knot has a functional IpaB domain and a folded Knot domain which indicates that the fusion protein is folded prior to secretion. However, the fusion protein attenuates invasion as observed for an *ipaB*-deficient mutant. In order to pass through the narrow NC channel, the T3SS needs to unfold the Knot. We observed that IpaB-Knot inhibits secretion of T3SS translocators and effectors in the hypersecretor *S. flexneri* 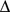 *ipaD*. Obstruction may occur because the T3SS cannot unfold the Knot domain within IpaB-Knot and consequently, IpaB-Knot blocks the channel. Our results are supported by previous reports which show a direct correlation between T3SS secretion and the ability of a protein to be thoroughly inserted into the channel and its folding status [17] [18].

As IpaB-Knot obstructed the effector secretion, we next analyzed the interaction between IpaB-Knot and NCs. Interaction was indicated as IpaB-Knot migrated with NCs in gradient fractionations. This co-migration was limited to endogenous IpaB-Knot and therefore takes place only inside the bacterium as it could not be restored by mixing purified IpaB-Knot with isolated NCs. This suggests an active mechanism mediating this interaction, probably also with the contribution of other factors. At this point, more experimental work is required to elucidate the underlying mechanism.

IpaB-Knot was detected with isolated NCs and this co-localization was shown to be specific. To our knowledge, this is the first visualization of a T3SS substrate together with the NC. Based on the N-terminal signal sequence and chaperone binding domains, the N-terminus of effectors provides a T3SS-specific secretion signal [21] [32]. We found that IpaB-Knot is at least partially secreted as its N-terminus could be detected at the tips of isolated NCs. This implies that, besides carrying the signal for secretion, the N-terminus of IpaB is also secreted first since our fusion does not allow secretion of IpaB by the C-terminus.

We show that the channel physically encloses the fusion protein. An embedded TEV protease-cleavage site in IpaB-TEV-Knot isolated with NCs was inaccessible to TEV protease. Furthermore, the protein is partially inaccessible to PEGylation. From these experiments we conclude that IpaB-TEV-Knot is secreted through the NC channel. This is supported by our observation that the interaction between the fusion and the NC is stable. We isolated the NCs with IpaB-TEV-Knot based on the interaction of a C-terminal Strep-tag in IpaB-TEV-Knot.

These results confirm the linear secretion model as well as secretion through the needle of the NC. We provide a tool for structural investigations of the NC in a stabilized active state and in association with a substrate protein. This allows the possibility to identify novel interaction partners for the T3SS substrates, which may still be associated with the complex at the stage where secretion is arrested. Also, the interaction of the substrate with structural components inside the channel can be studied in detail. This approach might also be used for other secretion systems, where proteins are required to unfold in order to be secreted. Ultimately, this contributes to the understanding of an essential mechanism of bacterial virulence which in turn might lead to novel antibacterial strategies.

## Materials and Methods

### Bacterial cultures, strains and plasmids

The bacterial strains and plasmids used in this study are listed in table 1. Bacterial cultures were grown in Tryptic Soy Broth (TSB) medium supplemented with antibiotics as selection markers. Strains were kept on TSB agar plates supplemented with antibiotics and 0.01% (w/v) Congo red (Sigma). Mutant strains were generated following the protocol from Datsenko and Wanner [33] using the pKD3 for chloramphenicol cassette amplification. The cassettes were flanked by homologous regions of the insertion sites which were included in the oligonucleotides for cassette amplification. For gene inactivation, the open reading frame was replaced with the antibiotic cassette in the bacterial genome. For fusion constructs, the fusion partner-gene was ligated to the antibiotic cassette by PCR and inserted into the genome. Oligonucleotides used for genetic modifications of bacteria are listed in table 2.

**Table 1:** Bacterial strains and plasmids used in this study.

bacterial strain	source
*Shigella flexneri* M90T	serovar 5a isolate
*S. flexneri* Δ*ipaD*	Lab collection [7]
*S. flexneri* Δ*mxiH*Δ*ipaD*	this study
*S. flexneri* Δ*ipaD*::*ipaBknot*	this study
*S. flexneri* Δ*ipaD*::*ipaAknot*	this study
*S. flexneri* Δ*ipaD*::*ipaH7.8knot*	this study
*S. flexneri* Δ*ipaD*::*ipaBTEVknot*	this study
*Escherichia coli* BL21 RIL	Novagen
plasmid backbone	insert
pASK-IBA3+	*ipaBknot*
pASK-IBA3+	*ipaBTEVknot*
pASK-IBA33+	*knot*
pET28a	*ipgC* [35]

Bacterial strains and expression plasmids used.

**Table 2:** Oligonucleotides for genetic modifications performed.

purpose	sequence (5′ to 3′)
*mxiH* deletion in the *ipaD* mutant:	
5′ chloramphenicol forward, flanking *mxiG*	AAA TAA GTG AGG ATA AAA TGA GTG TTA CAG TAC CGA ATG ATG ATT GGA CAA TGG GAA TTA GCC ATG GTC C
3′ chloramphenicol reverse, flanking *mxiI*	GAA TGC TCC CTA TTA TCT GAA GTT TTG AAT AAT TGC AGC ATC AAC ATC CTG TGT AGG CTG GAG CTG CTT C
*knot* insertion primer:	
5′ *knot* forward *ipaB*-fusion	AGC CTC AAT GTC CAA CTC TCA GGC TAA TAG AAC TGA TGT TGC AAA AGC AAT TTT GCA ACA AAC TAC TGC TAT GCG CAT TAC CTC AAC GGC G
5′ *knot* forward *ipaA*-fusion	AGC AAA AGA TGT AAC CAC TTC CCT ATC AAA AGT ATT AAA GAA TAT CAA TAA GGA TAT GCG CAT TAC CTC AAC GGC G
5′ *knot* forward *ipaH7.8*-fusion	CTG ACG AGG TAC TGG CCC TGC GAT TGT CTG AAA ACG GCT CAC GAC TGC ACC ATT CAA TGC GCA TTA CCT CAA CGG CGA AT
5′ *knot* forward, including *ipaC* RBS	TCC AAC TCT CAG GCT AAT AGA ACT GAT GTT GCA AAA GCA ATT TTG CAA CAA ACT ACT GCT TAA TAC AAA TAA GGA GAA TGT TAT GCG CAT TAC CTC AAC GGC
5′ *knot* forward *ipaB*-fusion, including TEV site	AGG CTA ATA GAA CTG ATG TTG CAA AAG CAA TTT TGC AAC AAA CTA CTG CTG AAA ACC TTT ATT TTC AGT CTA TGC GCA TTA CCT CAA CGG CG
3′ *knot* reverse, overlapping oligonucleotide for pKD3 5′ chloramphenicol amplification	GGA CCA TGG CTA ATT CCC ATT TAT TAT TTT TCG AAC TGC G
5′ chloramphenicol forward, overlapping oligonucleotide for *knot* reverse amplification	CGC AGT TCG AAA AAT AAT AAA TGG GAA TTA GCC ATG GTC C
3′ chloramphenicol reverse for *ipaB*-fusion, including *strep*-tag	GGA TAT ATC TGT ATA TAA AGT CTG GGT TGG TTT TGT GTT TTG AAT TTC CAT AAC ATT CTC CTT ATT TGT AGT GTA GGC TGG AGC TGC TTC
3′ chloramphenicol reverse for *ipaA*-fusion	ACG CTA TAC CAT AGC AAA AGG CCA CTA TTG ATA ATA TTT TTT CTT TTA TCA TAT TGT GTA GGC TGG AGC TGC TTC
3′ chloramphenicol reverse for *ipaH7.8*-fusion	TCA CAG TTT TTC CGG AGT CAA TCC GGT CTG CGG TTT ATG CTT ATG CGA CGT GAG TGT AGG CTG GAG CTG CTT C

Oligonucleotides used for either genetic fusions or gene deletions.

### Secretion assay

Bacteria were grown over night and cell density (OD) was measured by absorption at 600 nm. Culture aliquots corresponding to an optical density of 2/ml (about 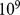 bacteria) were harvested and the supernatant was saved. Bacteria were washed once with phosphate buffered saline (PBS) and resuspended in 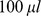 SDS sample buffer. The supernatant was filtered (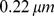) and proteins were precipitated with 10% (v/v) Trichloroacetic acid for 5 min at 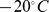 and harvested subsequently by centrifugation at 16.100 rcf for 30 min at 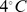. Pellets were washed once with 1 ml ice-cold acetone and centrifuged again for 30 min. Acetone was discarded directly after centrifugation and the pellets dried at room temperature and resuspended in 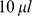 3 M TrisHCl, pH 8.5, plus 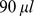 SDS sample buffer. Supernatants and 10% of the equivalent bacterial lysates were analyzed by SDS PAGE and Western blotting.

### Gentamicin protection assay

Invasion of *Shigella* strains was quantified as described previously [34]. 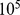 HeLa cells per well were seeded in 24-well plates and grown overnight in Dulbeccos Modified Eagle Medium (DMEM, Life Technologies)+10% fetal calf serum (FCS). Cell culture medium was exchanged the next day to DMEM without FCS (serum-free medium, SFM). HeLa cells were infected with 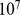 bacteria (log phase) in PBS for 30 min at 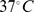 and centrifuged at 1.340 rcf for 10 min. Medium was exchanged to SFM+gentamicin to avoid continuous reinfection and bacteria were allowed to replicate inside cells for 2 h at 37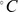. HeLa cells were then washed with PBS and lysed in the presence of 1% (v/v) Triton X-100 in PBS and bacteria were plated on LB agar. Bacterial invasion was quantified by number of colony-forming units (CFU) per ml culture after overnight incubation at 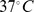.

### SDS PAGE and Western blot

All SDS PAGE (except 2D SDS PAGE) were performed using TrisHCl gradient gels (Criterion AnykD or 4–20%, Bio-Rad). Proteins were transferred on nitrocellulose membrane (GE Healthcare) with 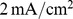 for 1 h and blocked with 5% (w/v) milk powder in PBS with 0.05% (v/v) Tween-20. Proteins were detected using monoclonal IpaB antibody (H16) [31], monoclonal MxiG antibody (7G1, this study), monoclonal DnaK antibody (Stressgen), monoclonal Strep-tag antibody (QIAGEN) or polyclonal IpaC antibody (Institut Pasteur). Visualization was performed using secondary antibodies coupled to horse radish peroxidase and enhanced chemiluminescence using a DuraStable West-Kit (Thermo Scientific).

### Purification of recombinant protein

IpaB-Knot or IpaB-Knot in complex with IpgC were purified as previously described [32] [35]. *E. coli* BL21 co-transformed with pET28a::*ipgC* (with a 3′ *6his*-tag) and pASK-IBA3+::*ipaBknot* (with a 3′ *strep*-tag) were grown to early log-phase and expression of *ipgC* was induced with 0.5 mM 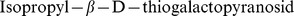 (IPTG, Thermo Fisher) for 30 min. Then, expression of *ipaBknot* or *ipaB* was induced with 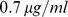 (w/v) anhydrotetracycline (Thermo Fisher). IpgC and IpaB-Knot was co-purified using 5 ml HisTrap HP cartridges (GE Healthcare) according to manufacturers instructions. The complex binds to the resin via IpgC and IpaB-Knot is eluted with 0.1% (w/v) Lauryldimethylamine N-oxide (LDAO). For fluorescence spectrometry and Proteinase K digestion, full complex of IpgC/IpaB-Knot was eluted with imidazole. Additional affinity chromatography in the presence of 0.05% LDAO using 5 ml Strep-Tactin columns (IBA) was performed to separate full length IpaB-Knot from IpaB contaminants which might occur because of instability of the fusion protein. Native protein was separated from aggregates by size exclusion chromatography using a Superdex 200 16/60 column (GE Healthcare, Freiburg) with 20 mM 4-(2-hydroxyethyl)-1-piperazineethanesulfonic acid (HEPES), pH 7.4, and 150 mM NaCl. The *knot* was expressed with a C-terminal His-tag (pASK-IBA33+::*knot*) in *E. coli* BL21 and purified via a 5 ml HisTrap HP column in 20 mM TrisHCl, pH 7.4, 300 mM NaCl, 1 mM Dithiothreitol (DTT), 40 mM Imidazole (ultra-pure) and eluted with 400 mM Imidazole under same buffer conditions. Proteins were concentrated to 1–2 mg/ml.

### Cytotoxicity assay

IpaB cytotoxicity was quantified as described before [25]. Murine bone marrow macrophages (mBMM) from C57BL/6J mice (Jackson Lab) were seeded in 96-well plates (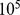 mBMM/well) in DMEM with 2% FCS and incubated overnight. Medium was replaced with DMEM with reduced FCS (0.5%). Different concentrations of recombinant IpaB-Knot or bovine serum albumin (BSA) were added to cells. The cells were then incubated for 2 h at 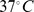. Supernatants were tested for LDH using a colorimetric assay (Promega) according to the manufacturers instructions.

### Fluorescence spectroscopy

Purified protein (either IpaB-Knot in complex with IpgC or Knot) was used at 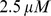 concentrations at 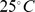. Samples were excited with 295 nm monochromatic light using 2.5 nm or 5 nm slit-width. Emission spectra were recorded at 300–400 nm at a slit-width of 5 nm or 10 nm. Control spectra (buffer with IpgC as a control for IpaB-Knot in complex with IpgC) were subtracted from sample protein spectra. Emissions were normalized to their respective maximum.

### Proteinase K digestion and mass spectrometry (MS)

Purified IpaB-Knot in complex with IpgC was treated with Proteinase K at a molar ratio of 1∶100 (enzyme to substrate) for 1 h at room temperature. The reaction was stopped by the addition of complete EDTA-free protease inhibitor cocktail (Roche) and the cleavage products were separated by 2D SDS PAGE. Respective protein cleavage products were trypsinated and analyzed by MS, whereas fragments of specific interest were confirmed by MS/MS.

### Isolation of needle complexes

Needle complexes were isolated from bacteria as described previously [12]. 2 L of bacterial culture were inoculated 1∶50 and grown to an optical density of 1.2–1.6/ml and washed once with PBS. Cells were osmotically shocked by resuspension in 0.5 M sucrose. 0.1 M TrisHCl, pH 8.0 and 5 mM EDTA were added. Bacteria were incubated in the presence of 1–2 mg lysozyme (Novagen) for 30–60 min and lysed with 2% (v/v) Triton X-100. Debris was removed by centrifugation for 20 min at 45.000 rcf and needle complexes were harvested by pelleting the supernatant at 110.000 rcf for 1 h. The pellet was washed in 10 mM TrisHCl pH 8.0, 150 mM KCl, 5 mM EDTA, 1% (v/v) Triton X-100 and 0.3% (w/v) Sarcosyl. Centrifugation steps were repeated and pelleted needle complexes were resuspended in 1–2 ml 50 mM TrisHCl, pH 8.0, 50 mM EDTA, 0.1% Triton X-100 (TET buffer) over night. Needle complexes were further separated either by CsCl gradient centrifugation (27.5% (w/v) CsCl in TET) and size exclusion chromatography (Superose 6 16/60, GE Healthcare, in TE buffer) or affinity purifaction via Strep-Tactin sepharose (IBA) in TET via the C-terminal Strep-tag of IpaB-Knot. Needle complexes were eluted with TET +5 mM desthiobiotin.

### Immuno-electron Microscopy (iEM)


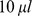
 aliquots of the needle complex preparations were applied to freshly glow-discharged carbon- and pioloform-film-coated copper grids and allowed to adsorb for 10 min. The grids were then washed with PBS, blocked for 15 min and incubated with mouse or human IpaB monoclonal antibody, anti-Strep antibody or both for 2–3 h at room temperature. After washing with PBS the samples were incubated with goat-anti-mouse antibody adsorbed to 12 nm gold particles for 30 min and washed with PBS. After three washes with distilled water, the grids were contrasted with 4% phospho-tungstic-acid (PTA), 1% Trehalose, pH 7.0, touched onto filter paper and air-dried. The grids were examined in a LEO 906 (Zeiss AG) electron microscope operated at 100 kV and images were recorded with a Morada (SIS-Olympus) digital camera.

### Nearest-neighbor analysis

Coordinates for gold labels and needle complex centers from 50 iEM images were manually obtained using the ImageJ analysis package [36]. The values were processed by R [37] and subjected to a nearest-neighbor analysis with the Biobase package [38].

### Protease protection assay

Isolated needle complexes or recombinant protein was added to 100 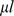 Strep-Tactin beads (IBA) and incubated for 1–2 h at 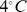 with gentle rotation (12–15 rpm). Beads were washed twice with 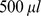 TET buffer and subsequently washed twice with 1 ml 50 mM Tris, pH 8.0, 0.5 mM EDTA, 1 mM DTT (TEV assay buffer) and finally resuspended with 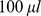 TEV assay buffer. 4 aliquots of 40 µl bead slurry were adjusted to room temperature and subsequently incubated with either 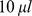 TEV assay buffer (negative control), 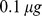, 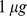 or 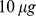 TEV protease at 400 rpm at 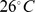. The beads were collected by centrifuging at 2000 rcf for 2 min. 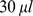 aliquots of supernatant were collected and supplemented with SDS sample buffer. The bead fraction was washed 2 times with 1 ml TEV assay buffer and the beads resuspended in 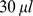 TEV assay buffer and supplemented with SDS sample buffer. Samples were analyzed by SDS PAGE and Western blotting.

### PEGylation assay

Isolated needle complexes or recombinant protein was added to 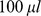 Strep-Tactin beads (IBA) and incubated for 1–2 h at 4°C with gentle rotation (12–15 rpm). Beads were washed twice with 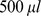 TET buffer and subsequently washed twice with PBS and finally resuspended with 50 µl PBS. 2 aliquots of 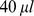 bead slurry were adjusted to room temperature and subsequently incubated with 0.5 mM PEGylation reagent MS(PEG)24 (Thermo Scientific) for 1 h at room temperature. The reaction was quenched with the addition of 100 mM TrisHCl for 5 min at room temperature and the reaction was directly incubated with SDS sample buffer and analyzed by SDS PAGE and Western blotting.

## Supporting Information

Figure S1 Qualitative analysis of purified IpaB-Knot (93 kDa) size-exclusion chromatography fractions by SDS PAGE and Coomassie stain.
(TIFF)

Figure S2 Mass spectrometry data of limited proteolysis. Schematic of the fusion is depicted as indicated with the trefoil-knot motif highlighted in green. Amino acid sequence of the knot with peptides detected by mass spectrometry in red and MS/MS-confirmed (red/bold).
(TIFF)

Figure S3 Effects of *knot* expression without effector fusion. (*A*) Supernatants of Δ*ipaD*::*knot*. (*B*) Western blots of supernatants and corresponding lysates using Strep-tag, DnaK or SepA antibodies.
(TIFF)

Figure S4 IpaB-labeling at NC base with anti-IpaB mouse monoclonal and 12 nm-gold conjugated anti-mouse antibody.
(TIFF)

Figure S5 Quantification of needle complexes (NC) and gold particles (Au) from immuno-EM. n = 50 images, black bars indicate mean.
(TIFF)
