# Supplementary figures and images for: A Substrate-Fusion Protein Is Trapped inside the Type III Secretion System Channel in Shigella flexneri
Source: PLoS Pathog. 2014 Jan 16;10(1):e1003881. doi: 10.1371/journal.ppat.1003881 (PMC3894212; doi:10.1371/journal.ppat.1003881)

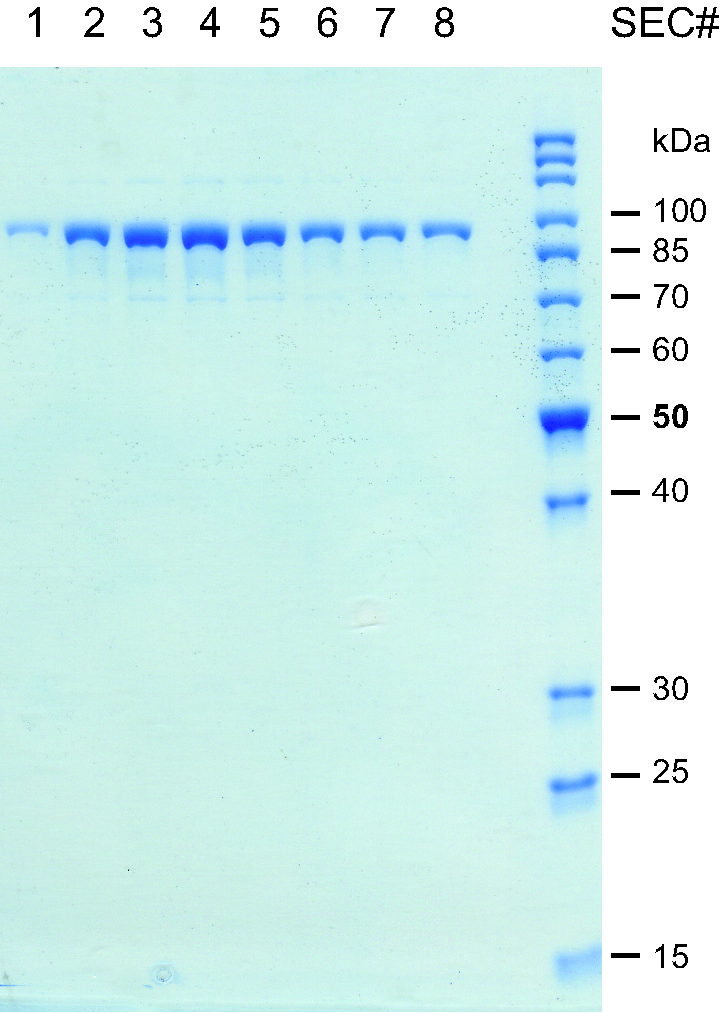

Supplement: Figure S1 — Qualitative analysis of purified IpaB-Knot (93 kDa) size-exclusion chromatography fractions by SDS PAGE and Coomassie stain. (TIFF) [file ppat.1003881.s001.tiff]

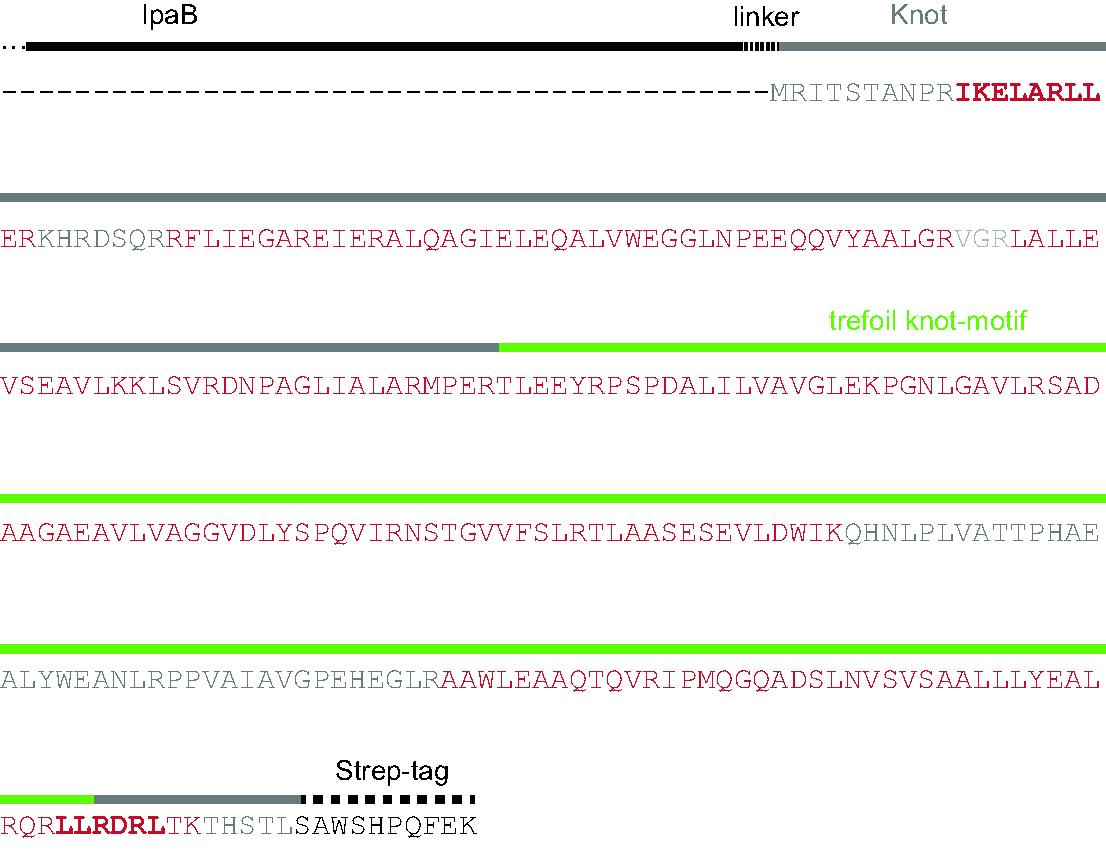

Supplement: Figure S2 — Mass spectrometry data of limited proteolysis. Schematic of the fusion is depicted as indicated with the trefoil-knot motif highlighted in green. Amino acid sequence of the knot with peptides detected by mass spectrometry in red and MS/MS-confirmed (red/bold). (TIFF) [file ppat.1003881.s002.tiff]

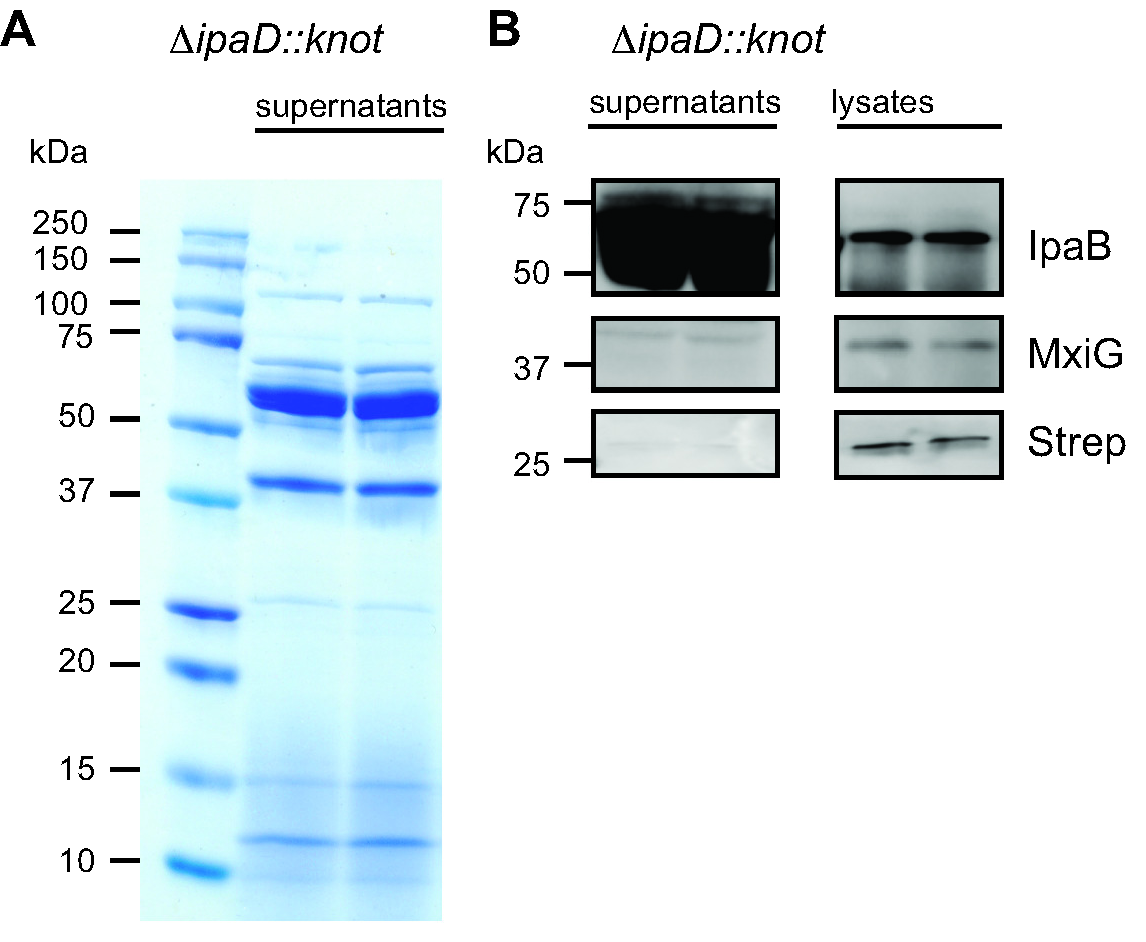

Supplement: Figure S3 — Effects of knot expression without effector fusion. (A) Supernatants of ΔipaD::knot. (B) Western blots of supernatants and corresponding lysates using Strep-tag, DnaK or SepA antibodies. (TIFF) [file ppat.1003881.s003.tiff]

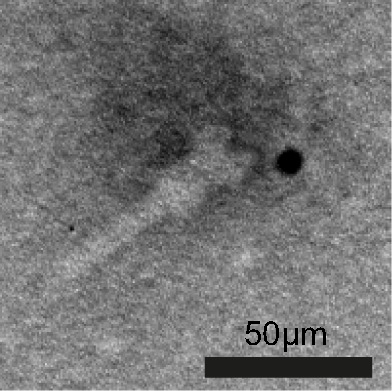

Supplement: Figure S4 — IpaB-labeling at NC base with anti-IpaB mouse monoclonal and 12 nm-gold conjugated anti-mouse antibody. (TIFF) [file ppat.1003881.s004.tiff]

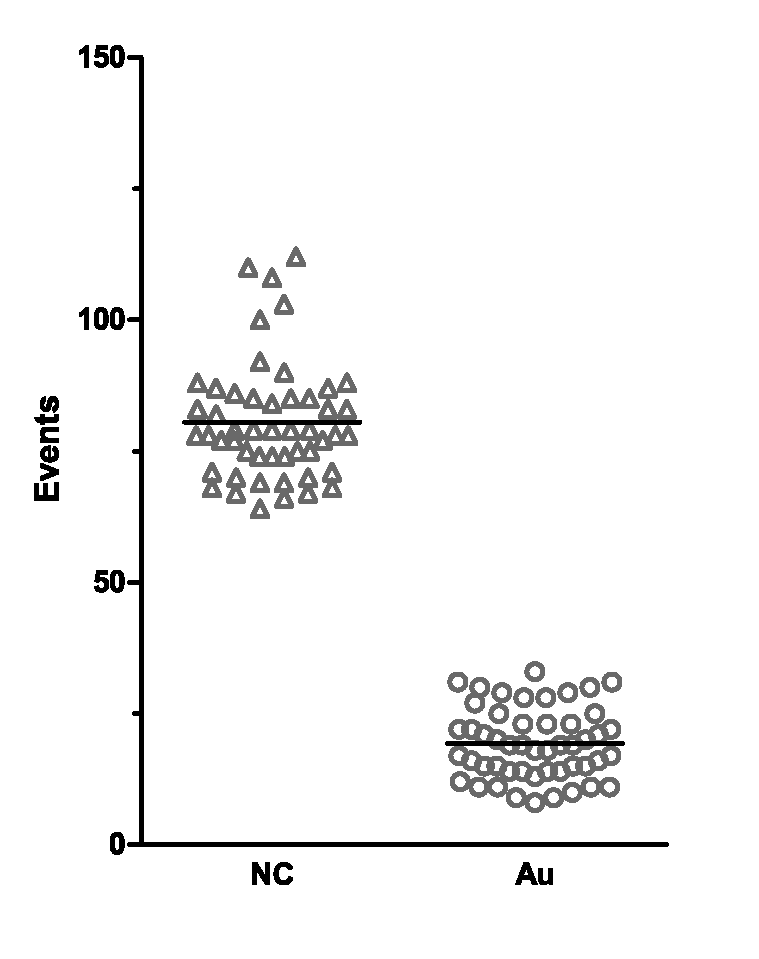

Supplement: Figure S5 — Quantification of needle complexes (NC) and gold particles (Au) from immuno-EM. n = 50 images, black bars indicate mean. (TIFF) [file ppat.1003881.s005.tiff]
